# Transitions and Resilience in Ecological Momentary Assessment: A Multiple Single-Case Study

**DOI:** 10.17505/jpor.2024.27102

**Published:** 2024-12-13

**Authors:** Merlijn Olthof, Andrea Bunge, Dominique F. Maciejewski, Fred Hasselman, Anna Lichtwarck-Aschoff

**Affiliations:** 1 Faculty of Behavioural and Social Sciences, University of Groningen, Groningen, The Netherlands; 2 Behavioural Science Institute, Radboud University, Nijmegen, The Netherlands; 3 Tilburg School of Social and Behavioral Sciences, Tilburg University, The Netherlands

**Keywords:** ecological momentary assessment, experience sampling, complex systems, non-stationarity, time series, resilience, transition, affect dynamics

## Abstract

Ecological momentary assessment (EMA) of affect, cognition and behavior aims to provide a ‘window into a person’s daily life’. But what should we look for through this window? In this paper, we compare a statistical perspective, grounded in probability theory, with a dynamic pattern perspective, grounded in complexity theory, on two common phenomena in EMA data: non-stationarity and outlying values. From a statistical perspective, these phenomena are considered nuisances that should be dealt with. From a dynamic pattern perspective, in contrast, non-stationarity may signal transitions from one dynamic pattern to another (e.g., a transition from a neutral to a persistent sad mood), whereas outlying values may signal recovery from perturbations (e.g., stressful life events). We evaluated the dynamic pattern view with a triangulation study of multiple single cases that took part in the Track your Mood EMA study, where participants reported on their emotions and daily events for 60 days. We found that non-stationarity was indeed related to a pattern transition, whereas outlying values were related to recovery after perturbations. These findings show that person-oriented EMA research would benefit from a dynamic pattern perspective that can identify highly meaningful and clinically relevant phenomena that are otherwise at risk of being missed. Complementing EMA time series with contextual information and qualitative data will be essential to genuinely understand these phenomena.

*It is the theory that decides what can be observed.* (Einstein as quoted by Heisenberg, 1971, pp. 62-63)

Ecological momentary assessment (EMA) and experience sampling methods, in which participants repeatedly answer questions through a smartphone app, are promising tools for person-oriented research in psychology. The hope of these methods is that intensive longitudinal measures of affect, cognition and behavior can provide a ‘window into a person’s daily life’. But what should we look for through this window? What are interesting phenomena and what is noise? In this paper, we compare a statistical perspective, grounded in probability theory, with a dynamic pattern perspective, grounded in complexity theory, on two common phenomena in EMA data: non-stationarity and outlying values.^1^ We show that what is considered noise in the statistical perspective can correspond to valuable information in the dynamic pattern view. We evaluate the dynamic pattern view with an analysis of multiple single cases that took part in the Track your Mood EMA study, where participants reported on their emotions and contextual information for 60 days (https://osf.io/fx3ay/).

The statistical perspective, grounded in probability theory, is currently dominant for analyzing EMA data. The key assumption in this perspective is that psychological phenomena can be studied by the estimation of statistical parameters, such as means, standard deviations, (lag-1) autocorrelation, cross-correlation and (lagged) conditional associations, which are assumed to be stable over time. For example, the standard deviation of EMA measures of affect is used as a measure of emotional variability, whereas the lag-1 autocorrelation of emotion items is used as a measure of inertia (Dejonckheere et al., 2019). Given certain assumptions, models with lagged associations can be used to study granger causality: whether the value of one variable predicts the value of another variable at a subsequent timepoint, correcting for all other associations (e.g., Hamaker & Wichers, 2017). This enables research from a statistical perspective to also study potential causal associations in observational EMA data, which is of high clinical relevance.

While dominant in the literature, the statistical perspective is not the only way to approach EMA data and hence not the only view one can have into ‘the window of a person’s daily life’. One alternative view, grounded in complex systems theory, is to examine EMA data as a measure of dynamic patterns (e.g., Hasselman & Bosman, 2020; Heinzel et al., 2014; Olthof et al., 2020; Wichers et al., 2020). In short, a dynamic pattern perspective assumes that a person’s mental state is a contextualized and self-organized pattern of cognitions, emotions and behaviors that is only dynamically stable (Hayes & Andrews, 2020; Olthof, Hasselman, Oude Maatman, et al., 2023). Persons can temporarily drift away from a dominant pattern (e.g., when a happy person has a ‘bad day’) and can experience transitions between qualitatively different patterns (e.g., from a depressed pattern towards a non-depressed pattern). Interestingly, the dynamic pattern view thereby provides a vastly different perspective on two phenomena in EMA data that are often considered methodological problems in the statistical perspective: non-stationarity and outlying values.

## Non-Stationarity

Non-stationarity can pertain to several different properties of a time series that do not remain constant over time (Molenaar, 2004). Most commonly, non-stationarity refers to the central moments of a distribution (e.g., mean, variance, kurtosis), but it could also refer to changes in the trend of the data, or in the autocorrelation function (Kelty-Stephen et al., 2022). When there is non-stationarity of one or more distributional properties, one cannot adequately summarize the EMA data of an individual in terms of a characteristic scale (mean, standard deviation) or a typical dynamical pattern (fluctuation intensity, periodicity). Statistical modelling of EMA data often assumes stationarity (Piccirillo & Rodebaugh, 2019). Researchers therefore try to “correct” for non-stationarity and for example try to remove non-stationarity of the mean by applying methods such as (polynomial) detrending.

From a dynamic pattern perspective, non-stationarity is not a methodological problem that one would like to get rid of, but a major research avenue as it may signal the *pattern transition* from one dynamic pattern to another (for example from a general positive mood to a depressed mood, or from a neutral mood to a pattern of frequent mood swings). Such pattern transitions have been extensively studied with EMA in psychotherapy and psychopathology research (Hayes & Andrews, 2020). In psychotherapy, pattern transitions in symptom severity (Helmich et al., 2020), or even personalized ratings (Olthof, Hasselman, Aas, et al., 2023), appear to be quite common and related to better treatment outcome. Researchers have also tried to predict such pattern transitions, with mixed results so far (Cui et al., 2022; Helmich et al., 2024). Notably, pattern transitions have also been studied in other areas of psychological science, such as movement science (Kelso et al., 1986), development (Thelen & Smit, 1994), cognition (Stephen et al., 2009) and team collaboration (Wiltshire et al., 2018).

## Outlying Values

From a statistical perspective, outlying values in EMA data present another nuisance that biases statistical inference. Similar to analyses in between-person statistics, outliers can skew the distribution, which is often assumed to be symmetrical (at least in the residuals). Therefore, outliers are sometimes winsorized (set to a certain percentile) or removed from EMA data.

From a dynamic pattern perspective, outliers are interesting because they could indicate moments in time at which a person was strongly deviating from their currently stable dynamic pattern. For instance, a person may generally have a positive mood but experience a specific moment of distress after which the person recovers, leading to a couple of outlying values of lower positive affect. Viewing this from a dynamic pattern perspective, we may ask ourselves: what kind of event (*perturbation* in system terms) caused this distress? And how *resilient* is the dynamic pattern of positive mood? Can a person quickly return to their positive mood pattern, or will feelings of distress linger on for a long time? Examining this *return time* (i.e., how long it takes to return to the dynamic pattern; Scholz et al., 1987) is an interesting research avenue for future resilience research (e.g., Vaessen et al., 2019).

## Current Study

In this study, we examined the dynamic pattern perspective on non-stationarity and outlying values (i.e., as indicators of pattern transitions and recovery after perturbation) by triangulation of time series of affect, time series of daily events and qualitative information from data viewing sessions with participants. We report on several cases from the Track Your Mood study, a 60-day EMA study. We studied whether (1) a pattern transition in momentary affect could be related to a shift in experience for the person as reported in the data viewing sessions and (2) outlying values in momentary affect could be related to recovery following specific events (perturbations), as reported in the daily event data and in the data viewing sessions.

## Method

### The Track Your Mood study

The Track Your Mood study allowed participants to rate their *mood* for 60 days and subsequently *track* how their emotional state developed over time. The Track your Mood study was approved by the ethical Committee of the Faculty of Social Sciences, Radboud University Nijmegen (Protocol number: ECSW-2021-075). Detailed information about the procedure of the project can be found in the Track your Mood project description at https://osf.io/fx3ay). To capture fluctuation of affect throughout each day, participants were asked to rate their mood at five separate timepoints (fixed schedule, e.g. 9:00, 12:00, 15:00, 18:00 and 21:00) by answering the question ‘*how do you feel at this moment?*’ (Medland et al., 2020). Participants answered this question by means of a 100-point sliding scale from ‘*very bad*’ to ‘*very good*’. At the end of the day, persons were additionally asked to provide contextual information by answering questions about their experienced emotions and events that occurred that day (for a comprehensive overview, see https://osf.io/vjdrn). In the current study, we examined answers on an open question about negative events as well as the rated intensity of the event. Participants were presented with the text “*Think of today’s most negative event*” and were asked to answer in an open box on the question “*What was it?*”. In addition, they were asked to rate the intensity of the event with the question “*This event was…”* from 0 (neutral) to 100 (very unpleasant).

All questions were sent to the participants through a mobile phone application ‘*m-path*’ (Mestdagh et al., 2022). The application was installed on the participant’s phone and configured together with the researchers at the beginning of the study. While all participants were asked the same questions and in the same intervals (5 times a day with a 3-hour buffer between questions), the exact times at which participants received notifications to answer their momentary affect questions were personalized based on the availability of the person in question.

In total, 77 participants between the ages of 18-53 (*M* = 22.32 years, *SD* = 5.98) completed the study. Throughout the entire study period six persons of the 83 initial sign-ups dropped out. The remaining 77 participants had an average compliance rate of 78%. Most participants were students attending Radboud University in Nijmegen, the Netherlands (92%) and had a mean age of 22.21 (*SD* = 6.06, range 18-53). Of these participants, 87% indicated that they identified as female, 12% as male, and 1% as non-binary. An exclusion criterium was that participants were only included if they were not in psychiatric or psychological treatment at the start of the study.

Of the 77 participants who completed the study, a subset of 31 signed up for data-viewing sessions after the 60 days EMA period. At this meeting, participants were able to view their own submitted data in overviews reports created with RMarkdown. This allowed participants to explore their data together with the researchers and ask questions. The data viewing conversations were primarily driven by what participants would bring up spontaneously and in response to specific graphs in their overview report. An overview report would feature graphs of the momentary affect time series with various additional visualizations such as linear trends, smoothed trends and changes between mean levels. In addition, graphs would illustrate the days of the 3 most negative and 3 most positive events projected on the time series of momentary affect. If the researchers noticed something they found interesting, but that was not mentioned by the participant, they would ask the participant. The researchers aimed to respond carefully and empathically to participants, being sensitive to the personal and emotional topics that came up in the conversations. The researchers avoided suggesting causal interpretations of data patterns and emphasized the descriptive and idiographic nature of participant’s graphs. Participants were offered to receive their overview report as a html file.

### Participants in the Multiple Single-Case Study

**Figure 1:**
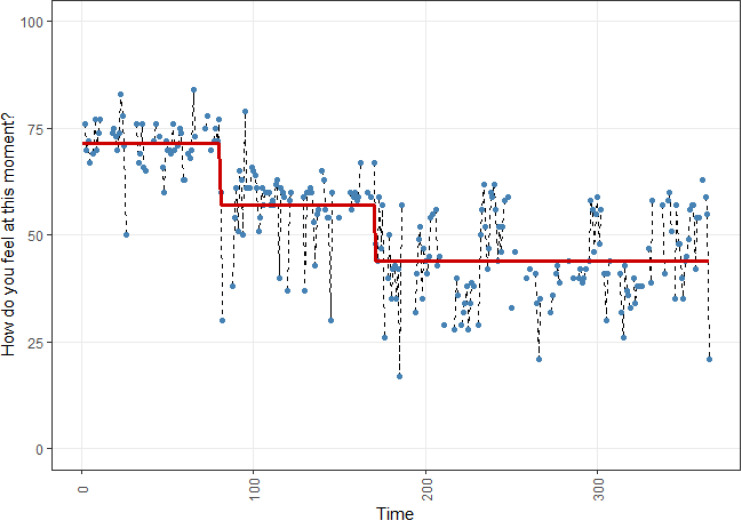
Affect dynamics of participant A.

**Figure 2:**
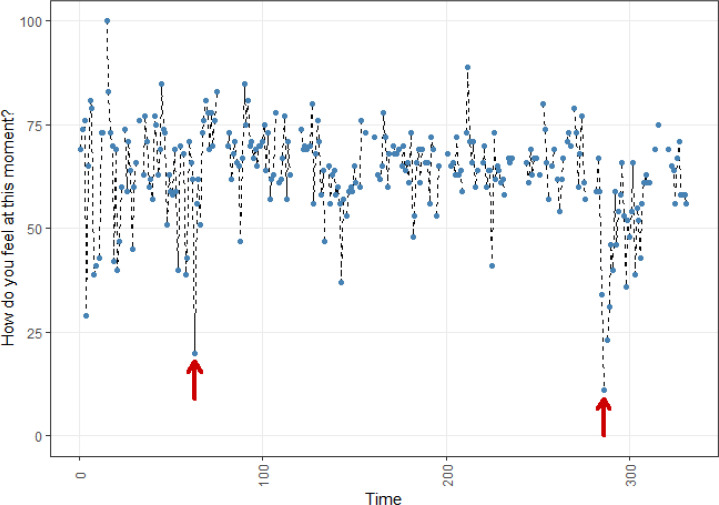
Affect dynamics of participant B

**Figure 3:**
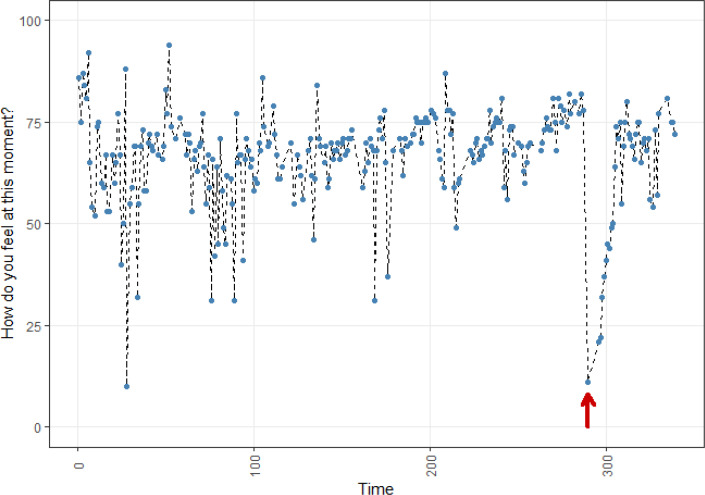
Affect dynamics of participant C

**Figure 4:**
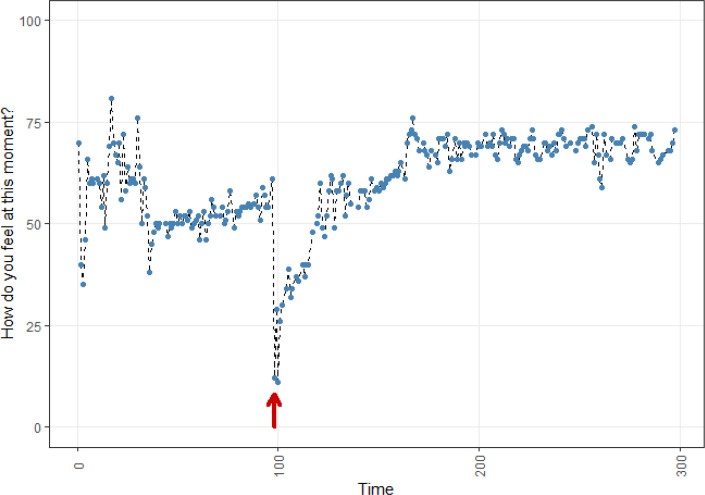
Affect dynamics of participant D.

**Figure 5:**
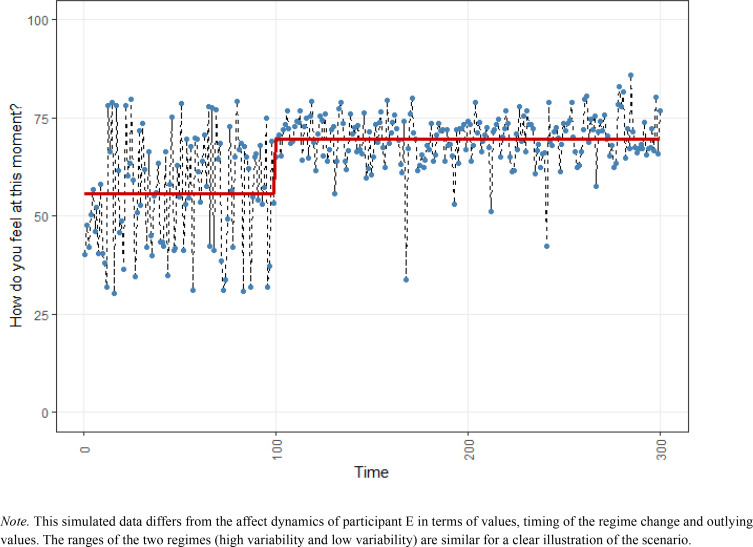
Simulated affect dynamics illustrating the data pattern of participant E.

Participants were considered for the current study if they had high compliance on the EMA assessment (>=80%) and participated in the data viewing session. Our general strategy was to first identify cases of non-stationarity and outlying values based on the affect time series and then use the daily event data and the notes from the data viewing sessions to evaluate a dynamic pattern perspective on these cases. For participant E (see below), the strategy is different, because we remembered her case vividly and we decided to include her as an example of how experience and data may not align. In case of non-stationarity, recursive partitioning (described below) was used to classify stable levels and transitions between these levels (see figure 1 and 5). Recovery after outlying values was identified by visual inspection (see Figure 2-4).

We report first on one participant (participant A; a woman in her early 20s) in our sample that showed clear non-stationarity (potential pattern transitions) in her data, had high compliance (80%) on the affect and the daily event measures and spoke with us in the data viewing session. There were no other participants with clear pattern transitions in the momentary affect data that we also spoke with in the data viewing session. We further report on three exemplar participants (participants B-D, a man is his late teens and two women in their early 20s) who showed a clear pattern of recovery from perturbation, had high compliance (88% - 90%) in the affect and daily event measures and spoke with us in the data viewing session. These exemplars all show recovery after a perturbation, but with different trajectories, which we interpret in relation to the daily events and subjective experiences. To highlight that patterns in the data did not always match participants’ experience, we also included the counterexample of a participant (participant E, a woman in her early 20s) who explained to us in the data viewing session how her time series were not valid for a part of the data collection period (compliance 88%). The median time between the end of the EMA assessment and the data viewing session for the 6 participants was 20 days (range 18-31).

### Recursive Partitioning

Pattern transitions in momentary affect were classified by recursive partitioning, as used in previous studies (Olthof et al., 2020; Olthof, Hasselman, Aas, et al., 2023). Recursive partitioning seeks to optimally split a time series into segments that maximally differ from each other. We used the function *ts_levels()* in the R-package *casnet*, which in turn calls the function *rpart()* from the *rpart package*, and used ANOVAs to evaluate the splits. If the split leads to an *R^2^* increase of .10, the split is included and the algorithm searches for new optimal splits in the two segments. When the *R^2^* increase is smaller than .10, there will be no split. With this method and criterion, a time series can thus be split into two or more segments, but also have no split at all when the mean and variance are stable. This is why pattern transitions as identified with recursive partitioning are also indicative for non-stationarity in mean and variance.

## Results

### Non-Stationarity as Pattern Transition

Figure 1 shows the momentary affect time series of participant A. The red line shows three mean level patterns and the two shifts between them as classified by recursive partitioning. The time series unambiguously illustrate non-stationarity of the mean. The second shift was clearly related to A’s experience. She indicated that she felt very different and much more down from one day to another. She could not think of any reason why this had happened; according to her there was no clear environmental trigger. She found the first shift interesting, telling us that she did not recall experiencing a clear-cut shift there. For this shift it is thus unknown if she experienced a pattern transition in affect, or whether she started to use the measurement scale differently without being aware of it (Barta et al., 2012). During the data viewing session that took place about 3 weeks after the data collection finished, she told us that she was still on the ‘third level of her graph’, or perhaps on an ‘even lower (fourth) one’, and was starting psychotherapy.

### Outlier as Perturbation

Figure 2-4 show the momentary affect time series for participants B, C, and D. Participant B had two clear negative outlying values, which coincided with two negative events which he rated as most intense. For the first negative outlier, he reported having watched a sad television series. One can see that his affect recovers quickly within the interval to the next measurement point. At the second negative outlier, he reported that he had abdominal pains and had slept badly. Here, he recovers over the course of about 5 assessment to a more stable dynamic pattern, albeit characterized by slightly lower positive affect than the previous pattern. At the end of data collection, he appears to have reached his previous level of positive affect again.

Participant C told us that at the outlying value at the end of the time series a close relative of her partner deceased. She told us that it took her about a week to recover to some extent, which one can also see in the graph.

Participant D became ill at the negative outlying value and she told us that it took quite some time to recover for her. Indeed, one can see that she slowly recovered over the course of weeks to a stable level of relatively high positive affect. She was not aware of these relatively high scores at the end of her data collection. She was happy to see her data over-view report and took the data pattern as an indicator that she recovered well.

### Counter Example: When Data and Experience do not Align

In the data viewing session, participant E warned us that her time series were not representative for her affect at the end of the study period. In the later part of the data collection she had felt less positive than before. She told us that not her scores, but *the way in which she scored* was indicative of her affect. When feeling less positive, she always scored ‘a bit medium’, with low variability in her scores. Hence, the low variability in the later part of the data collection actually indicates more negative affect, according to the participant. Interestingly, at the point where her affect scores get less variable and she feels *less well*, the recursive partitioning algorithm showed a possible shift towards higher positive affect (Figure 5). Without the data viewing session, we could thus have falsely interpreted this as a transition towards more positive affect. Participant E did not give consent to publish her affect dynamics time series. Instead, we simulated data to illustrate this kind of pattern (Figure 5; note that the pattern transition was identified in the real data).

## Discussion

The exemplar cases in this study show that non-stationarity and outliers, often considered methodological problems from a statistical perspective, can signal pattern transitions, perturbations and recovery, which are highly relevant processes in a dynamic pattern view on affect dynamics. Participant A experienced a pattern transition towards feeling more down which eventually made her seek psycho-therapeutic help. She felt more down from one day to another and could not identify a clear cause for this transition. From a dynamic pattern view, we can further investigate this change process by evaluating different transition mechanisms (Cui et al., 2024; Hulsmans et al., 2024). For instance, in an event-induced transition, we would have expected to find a strong event as cause of the transition. The finding that such an event was absent in this case points to a bifurcation-induced transition, where a mixture of processes had made the previous (more positive) affective pattern unstable (Olthof, Hasselman, Oude Maatman, et al., 2023). When there is such instability, very minor events can function as ‘the straw that broke the camel’s back’. A bifurcation mechanism is thus a possible explanation for how such a sudden and large transition in affect can appear in the absence of a strong environmental trigger.

Participant B, C and D all exhibited recovery after perturbation, but at different paces and with different perturbations. Participant B felt temporarily more negative after watching a sad TV series but recovered within hours. Later, participant B experienced abdominal pains and a night of bad sleep, from which it took about a day to recover to some extent and then a few days more to bounce back to his previous level of positive affect. Participant C had to deal with the tragic loss of a close relative of her partner, from which she partly recovered over the course of a week. Participant D had an illness from which it took multiple weeks to recover. Interestingly, this participant did not recover to the pre-illness pattern but seemed to reach a whole different pattern of relatively high positive affect ratings.

The examples of participants B, C and D show that outlying values can be related to events that temporarily drive persons away from their current affective pattern. The recovery that often follows such perturbations illustrates the resilience of the affective patterns, which we can understand from complex systems theory by the notion of attractors. Attractors are dynamic patterns that a system keeps being ‘pulled towards’, even when the patterns are perturbed. For example, when there is an attractor of relatively positive affect, a person can experience momentary sadness (for example after watching a sad TV series, like participant B did), but will rather quickly return to the attractor again. Recovery times can therefore be informative about attractor strength: the faster one recovers from a perturbation, the stronger the attractor may be. For healthy attractors, the attractor strength is considered positive and related to the concept of resilience, while for psychopathological attractors, the attractor strength is part of the problem and can make people ‘stuck’ (Hayes et al., 2007; Olthof, Hasselman, Oude Maatman, et al., 2023; Schiepek et al., 2016).

It is, however, crucial to realize that recovery times are also related to the nature of the perturbations. Of course, one generally recovers faster from watching a sad TV series than from the loss of a family member. It is only in experimental settings, where one can provide controlled perturbations that one can learn something about the *current* resilience of a dynamic pattern (Thelen et al., 1991). Also, natural perturbations that are somewhat controlled, such as the moment when students receive their exam grades, may be used to study resilience processes (Baretta et al., 2023; Kalokerinos et al., 2023). But again, it is important to be cautious, as the same controlled perturbations may be experienced differently by different individuals and are thus only informative about specific forms of resilience (resilience to that specific perturbation). For example, an exam may be more perturbing (stressful) for person A than for person B, while for person B a visit from their parents-in-law may be more perturbing (stressful) than it is for person A.

A last issue to discuss is that persons not always return to the same attractor after perturbation but can also transition towards a different one. Indeed, our cases also show that recovery does not necessarily result in the same dynamic pattern as one had before (for example in participant D). This illustrates the importance of post-stressor change (Hill et al., 2024), besides recovery. Post-stressor change describes how the developmental trajectory of an individual may be changed in the long run by the perturbation. Hill et al. (2024) discuss the example that repeated exercise perturbs the muscles, which does not only lead to muscle recovery, but to growth. Post-stressor change can also be undesirable, for example in the case of post-traumatic stress, where specific perturbations have a long-lasting negative effect on a person.

While we contrasted a dynamic pattern perspective with a statistical perspective on EMA time series, it is important to realize that a dynamic pattern perspective is not necessarily incompatible with statistical *methods*. In fact, the recursive partitioning method that we used in this paper is based on statistical methods such as regression trees and ANOVA. Thus, also from a dynamic pattern perspective, it is exciting to see many novel developments in longitudinal statistical methods that are moving beyond stationary time series models such as change point analyses, time-varying models, hidden Markov models and more (e.g., Bringmann et al., 2017; Grip & Bergman, 2016; Hamaker et al., 2016). Whether research enacts a dynamic pattern perspective or a statistical perspective is not necessarily defined by specific methods, but by the assumptions researchers make (e.g., stationarity) and how they interpret results (i.e., as revealing time invariant properties of persons vs. as descriptions of processes). Central to this differentiation is how researchers approach the very notion of (time-varying) within-person variability: as noise or as process (Hasselman, 2023; Van Geert & Van Dijk, 2002; Van Geert & De Ruiter, 2022).

### Strengths and Limitations

A key strength of this study is the triangulation of EMA data, daily context measures and notes from the data viewing session to validate pattern transitions and recovery from perturbation. The daily context measures gave information about important events, thereby providing a fine-grained measure of perturbations. The data viewing sessions have proven to be crucial for valid interpretation of the EMA data (see also; De Smet et al., 2024; Truijens et al., 2021). Our participant E illustrates this point as her data and experience did not align and even showed opposite patterns (when the data stabilize at *increased* positive affect, she experiences *decreased* positive affect).

For the pattern transitions, the qualitative information that we got out of the data viewing sessions is very valuable from a complex systems theory point of view. Even the most fundamental transition theories in physics highlight that a pattern transition is first and foremost a *qualitative shift* in the behavior of a system (Haken, 1983). For instance, when liquid water transitions into ice, it is not only the order of the system that changes (which can be depicted in a time series of entropy values), but the *qualities* of the system change as well: ice has the quality that one may stand on it (when strong enough), while liquid water has totally different qualities (it can flow). In the case of affect transitions, complexity theory thus does not only predict a change in affect scores, but also a different quality in experience of affect, which we can best approach with qualitative data and methodology (see also, Schiepek et al., 2016).

In that respect, it is important to note that although we performed a very basic interpretative analysis of the text and conversation data, this study lacks the qualitative richness of a full mixed-methods approach (Hesse-Biber, 2010). While the current study is well-suited to examine our key hypotheses surrounding transitions and resilience in EMA, more interpretative work remains to be done in EMA research (De Smet, 2024). For example, mixed-methods research can also be used to study how participants use measurement scales (e.g., with think aloud protocols), how they come to certain answers and how they relate meaningfully to their own EMA data, for instance in terms of narrative identity, which all may contribute to a richer understanding of the phenomena of pattern transitions and resilience as well.

A limitation of this study is that it cannot shed light on the relative frequencies by which pattern transitions and recovery after perturbation are expected to occur in an EMA dataset. As a multiple case study, our aim was to study whether certain phenomena exist, but not how often they take place. This also means that we do not make any generalized claim that every instance of non-stationarity signals pattern transitions nor that every outlying value presents a meaningful perturbation that allows for studying recovery processes. However, we do pose that one cannot safely assume that non-stationarity and outlying values are simply uninformative sources of noise that should be corrected for, as one tends to do from a purely statistical perspective. As pattern transitions and recovery after perturbation are generic phenomena in all living systems (e.g., Scheffer et al., 2018), we would be very surprised if other EMA datasets do not contain them.

### Conclusion

This study illustrates that it is indeed ‘*the theory that decides what we observe*’ when we use EMA as a ‘window into a person’s life’. Where probability theory sees non-stationarity and outliers, complex systems theory sees pattern transitions and recovery from perturbations. In this study, we illustrated some exemplars in which further evidence for pattern transitions and recovery from perturbation was found in context measures and data viewing sessions. These findings show that person-oriented EMA research would benefit from broadening its scope beyond the statistical perspective and use a dynamic pattern perspective to identify highly meaningful and clinically relevant phenomena that are otherwise at risk of being missed. Complementing time series with contextual information and qualitative data will be essential to genuinely understand these phenomena.

## Data Availability

For inquiries about data availability, please contact the corresponding author.

## Footnotes

1 The distinction between these two perspectives closely matches the distinctions between substance and process ontology (Van Geert & de Ruiter, 2022) and between component-dominant and interaction-dominant dynamics (Van Orden et al., 2011). EMA research grounded in probability theory assumes component-dominant dynamics and enacts a substance ontology, whereas EMA research grounded in complexity theory assumes interaction-dominant dynamics and enacts a process ontology.

## Action editor

Lars-Gunnar Lundh served as action editor for this article.

## Open access

This article is distributed under the terms of the Creative Commons Attribution 4.0 International License (http://creativecommons.org/licenses/by/4.0/), which permits unrestricted use, distribution, and reproduction in any medium, provided you give appropriate credit to the original author(s) and the source, provide a link to the Creative Commons license, and indicate if changes were made.

## References

[cit0001] Baretta, D., Koch, S., Cobo, I., Castaño-Vinyals, G., De Cid, R., Carreras, A., Buekers, J., Garcia-Aymerich, J., Inauen, J., & Chevance, G. (2023). Resilience characterized and quantified from physical activity data: A tutorial in R. *Psychology of Sport and Exercise*, 65, 102361. 10.1016/j.psychsport.2022.102361 37665834

[cit0002] Barta, W. D., Tennen, H., & Litt, M. D. (2012). Measurement reactivity in diary research. In M. R. Mehl & T. S. Conner (Eds.), *Handbook of research methods for studying daily life* (pp. 108–123). The Guilford Press.

[cit0003] Bringmann, L. F., Hamaker, E. L., Vigo, D. E., Aubert, A., Borsboom, D., & Tuerlinckx, F. (2017). Changing dynamics: Time-varying autoregressive models using generalized additive modeling. *Psychological Methods*, 22(3), 409–425. 10.1037/met0000085 27668421

[cit0004] Cui, J., Hasselman, F., Olthof, M., & Lichtwarck-Aschoff, A. (2022). *Illuminating the path: Examining the research methods of early warning signals in clinical psychology through a theoretical lens*. 10.17605/OSF.IO/F659U PMC11924765 40108573

[cit0005] Cui, J., Hasselman, F., Olthof, M., & Lichtwarck-Aschoff, A. (2024). *Specifying transition types and scales of interest in understanding clinical change*. PsyArxiv. 10.31234/osf.io/b68wh

[cit0006] Dejonckheere, E., Mestdagh, M., Houben, M., Rutten, I., Sels, L., Kuppens, P., & Tuerlinckx, F. (2019). Complex affect dynamics add limited information to the prediction of psychological well-being. *Nature Human Behaviour*, 3(5), 478–491. 10.1038/s41562-019-0555-0 30988484

[cit0007] De Smet, M. M. (2024). Why (not) rely on patients’ perspectives? Time for a meaningful standard to measure and define patient-specific change in clinical psychology. *Clinical Psychology: Science and Practice*, 31(3), 370–374. 10.1037/cps0000213

[cit0008] De Smet, M.M., Imaani Quintens, L., Debeer, D., Van Nieuwenhove, K., Jongerling, J., & Meganck, R. (2024). *A mixed methods idiographic network approach for adolescent-specific assessment and care: A proof of concept*. ResearchGate. 10.13140/RG.2.2.20975.85929

[cit0009] Grip, A., & Bergman, L. R. (2016). A nonlinear dynamic model applied to data with two times of measurement. *Journal for Person-Oriented Research*, 2(1–2), 56–63. 10.17505/jpor.2016.06

[cit0010] Haken, H. (1983). *Synergetics: an introduction. Non-equilibrium phase transition and self-organisation in physics, chemistry and biology*. Springer Verlag.

[cit0011] Hamaker, E. L., Grasman, R. P. P. P., & Kamphuis, J. H. (2016). Modeling BAS dysregulation in bipolar disorder: Illustrating the potential of time series analysis. *Assessment*, 23(4), 436–446. 10.1177/1073191116632339 26906639

[cit0012] Hamaker, E. L., & Wichers, M. (2017). No time like the present: Discovering the hidden dynamics in intensive longitudinal data. *Current Directions in Psychological Science*, 26(1), 10–15. 10.1177/0963721416666518

[cit0013] Hasselman, F. (2023). Understanding the complexity of individual developmental pathways: A primer on metaphors, models, and methods to study resilience in development. *Development and Psychopathology*, 35, 2186–2198. 10.1017/S0954579423001281 37814420

[cit0014] Hasselman, F., & Bosman, A. M. T. (2020). Studying complex adaptive systems with internal states: A recurrence network approach to the analysis of multivariate time-series data representing self-reports of human experience. *Frontiers in Applied Mathematics and Statistics*, 6, 9. 10.3389/fams.2020.00009

[cit0015] Hayes, A. M., & Andrews, L. A. (2020). A complex systems approach to the study of change in psychotherapy. *BMC Medicine*, 18(1), 197. 10.1186/s12916-020-01662-2 32660557 PMC7359463

[cit0016] Hayes, A. M., Laurenceau, J.-P., Feldman, G., Strauss, J. L., & Cardaciotto, L. (2007). Change is not always linear: The study of nonlinear and discontinuous patterns of change in psycho-therapy. *Clinical Psychology Review*, 27(6), 715–723. 10.1016/j.cpr.2007.01.008 17316941 PMC3163164

[cit0017] Heisenberg, W. (1971). *Physics and beyond* (p. 129). Allen & Unwin.

[cit0018] Helmich, M. A., Schreuder, M. J., Bringmann, L. F., Riese, H., Snippe, E., & Smit, A. C. (2024). Slow down and be critical before using early warning signals in psychopathology. *Nature Reviews Psychology*, 3(11), 767–780. 10.1038/s44159-024-00369-y

[cit0019] Helmich, M. A., Wichers, M., Olthof, M., Strunk, G., Aas, B., Aichhorn, W., Schiepek, G., & Snippe, E. (2020). Sudden gains in day-to-day change: Revealing nonlinear patterns of individual improvement in depression. *Journal of Consulting and Clinical Psychology*, 88(2), 119–127. 10.1037/ccp0000469 31894994

[cit0020] Hesse-Biber, S. N. (2010). *Mixed methods research: Merging theory with practice*. Guilford Press.

[cit0021] Heinzel, S., Tominschek, I., & Schiepek, G. K. (2014). Dynamic patterns in psychotherapy – Discontinuous changes and critical instabilities during the treatment of obsessive-compulsive disorder. *Nonlinear Dynamics, Psychology and Life Sciences*, 8(2), 155–176. 24560009

[cit0022] Hulsmans, D. H. G., Otten, R., Poelen, E. A. P., van Vonderen, A., Daalmans, S., Hasselman, F., Olthof, M., & Lichtwarck-Aschoff, A. (2024). A complex systems perspective on chronic aggression and self-injury: Case study of a woman with mild intellectual disability and borderline personality disorder. *BMC Psychiatry*. 10.21203/rs.3.rs-3358763/v1 PMC11110386 38773533

[cit0023] Kalokerinos, E. K., Moeck, E. K., Rummens, K., Meers, K., & Mestdagh, M. (2023). Ready for the worst? Negative affect in anticipation of a stressor does not protect against affective reactivity. *Journal of Personality*, 91(5), 1123–1139. 10.1111/jopy.12787 36271680

[cit0024] Kelso, J. A. S., Scholz, J. P., & Schöner, G. (1986). Nonequilibrium phase transitions in coordinated biological motion: Critical fluctuations. *Physics Letters A*, 118(6), 279–284. 10.1016/0375-9601(86)90359-2

[cit0025] Kelty-Stephen, D. G., Lane, E., Bloomfield, L., & Mangalam, M. (2022). Multifractal test for nonlinearity of interactions across scales in time series. *Behavior Research Methods*, 55(5), 2249–2282. 10.3758/s13428-022-01866-9 35854196

[cit0026] Medland, H., De France, K., Hollenstein, T., Mussoff, D., & Koval, P. (2020). Regulating emotion systems in everyday life. *European Journal of Psychological Assessment*, 36. 10.1027/1015-5759/a000595

[cit0027] Mestdagh, M., Verdonck, S., Piot, M., Niemeijer, K., Tuerlinckx, F., Kuppens, P., & Dejonckheere, E. (2022). *m-Path: An easy-to-use and flexible platform for ecological momentary assessment and intervention in behavioral research and clinical practice*. PsyArXiv. 10.31234/osf.io/uqdfs PMC10619650 37920867

[cit0028] Molenaar, P. C. M. (2004). A manifesto on psychology as idiographic science: Bringing the person back into scientific psychology, this time forever. *Measurement: Interdisciplinary Research & Perspective*, 2(4), 201–218. 10.1207/s15366359mea0204_1

[cit0029] Olthof, M., Hasselman, F., Aas, B., Lamoth, D., Scholz, S., Daniels-Wredenhagen, N., Goldbeck, F., Weinans, E., Strunk, G., Schiepek, G., Bosman, A. M. T., & Lichtwarck-Aschoff, A. (2023). The best of both worlds? General principles of psychopathology in personalized assessment. *Journal of Psychopathology and Clinical Science*, 132(7), 808–819. 10.1037/abn0000858.supp 37843539

[cit0030] Olthof, M., Hasselman, F., & Lichtwarck-Aschoff, A. (2020). Complexity in psychological self-ratings: Implications for research and practice. *BMC Medicine*, 18(1), 317. 10.1186/s12916-020-01727-2 33028317 PMC7542948

[cit0031] Olthof, M., Hasselman, F., Oude Maatman, F., Bosman, A. M. T., & Lichtwarck-Aschoff, A. (2023). Complexity theory of psychopathology. *Journal of Psychopathology and Clinical Science*, 132(3), 314–323. 10.1037/abn0000740 37126062

[cit0032] Olthof, M., Hasselman, F., Strunk, G., Van Rooij, M., Aas, B., Helmich, M. A., Schiepek, G., & Lichtwarck-Aschoff, A. (2020). Critical fluctuations as an early-warning signal for sudden gains and losses in patients receiving psychotherapy for mood disorders. *Clinical Psychological Science*, 8(1), 25–35. 10.1177/2167702619865969

[cit0033] Scheffer, M., Bolhuis, J. E., Borsboom, D., Buchman, T. G., Gijzel, S. M. W., Goulson, D., Kammenga, J. E., Kemp, B., Van De Leemput, I. A., Levin, S., Martin, C. M., Melis, R. J. F., Van Nes, E. H., Romero, L. M., & Olde Rikkert, M. G. M. (2018). Quantifying resilience of humans and other animals. *Proceedings of the National Academy of Sciences*, 115(47), 11883–11890. 10.1073/pnas.1810630115 PMC6255191 30373844

[cit0034] Schiepek, G., Eckert, H., Aas, B., Wallot, S., & Wallot, A. (2016). *Integrative psychotherapy: A feedback-driven dynamic systems approach*. Hogrefe Publishing GmbH.

[cit0035] Schiepek, G. K., Stöger-Schmidinger, B., Aichhorn, W., Schöller, H., & Aas, B. (2016). Systemic case formulation, individualized process monitoring, and state dynamics in a case of dissociative identity disorder. *Frontiers in Psychology*, 7. 10.3389/fpsyg.2016.01545 PMC5072376 27812338

[cit0036] Scholz, J. P., Kelso, J. A. S., & Schöner, G. (1987). Nonequilibrium phase transitions in coordinated biological motion: Critical slowing down and switching time. *Physics Letters A*, 123(8), 390–394. 10.1016/0375-9601(87)90038-7

[cit0037] Stephen, D. G., Dixon, J. A., & Isenhower, R. W. (2009). Dynamics of representational change: Entropy, action, and cognition. *Journal of Experimental Psychology: Human Perception and Performance*, 35(6), 1811–1832. 10.1037/a0014510 19968438

[cit0038] Thelen, E., Ulrich, B. D., & Wolff, P. H. (1991). Hidden skills: A dynamic systems analysis of treadmill stepping during the first year. *Monographs of the Society for Research in Child Development*, 56(1), i. 10.2307/1166099 1922136

[cit0039] Truijens, F., De Smet, M. M., Desmet, M., & Meganck, R. (2021). Validity of data as precondition for evidence: A methodological analysis of what is taken to count as evidence in psychotherapy research. *Philosophy, Psychiatry, & Psychology*, 28(2), 115–128. 10.1353/ppp.2021.0018

[cit0040] Vaessen, T., Viechtbauer, W., Van Der Steen, Y., Gayer-Anderson, C., Kempton, M. J., Valmaggia, L., McGuire, P., Murray, R., Garety, P., Wykes, T., Morgan, C., Lataster, T., Lataster, J., Collip, D., Hernaus, D., Kasanova, Z., Delespaul, P., Oorschot, M., Claes, S., … Myin-Germeys, I. (2019). Recovery from daily-life stressors in early and chronic psychosis. *Schizophrenia Research*, 213, 32–39. 10.1016/j.schres.2019.03.011 30930036

[cit0041] Van Geert, P., & de Ruiter, N. (2022). *Toward a process approach in psychology: Stepping into Heraclitus’ river*. Cambridge University Press.

[cit0042] Van Geert, P., & Van Dijk, M. (2002). Focus on variability: New tools to study intra-individual variability in developmental data. *Infant Behavior and Development*, 25(4), 340–374. 10.1016/S0163-6383(02)00140-6

[cit0043] Van Orden, G. C., Kloos, H., & Wallot, S. (2011). Living in the Pink. In *Philosophy of Complex Systems* (pp. 629–672). Elsevier. 10.1016/B978-0-444-52076-0.50022-5

[cit0044] Wiltshire, T. J., Butner, J. E., & Fiore, S. M. (2018). Problem‐Solving phase transitions during team collaboration. *Cognitive Science*, 42(1), 129–167. 10.1111/cogs.12482 28213928

[cit0045] Wichers, M., Smit, A. C., & Snippe, E. (2020). Early warning signals based on momentary affect dynamics can expose nearby transitions in depression: A confirmatory single-subject time-series study. *Journal for Person-Oriented Research*, 6(1), 1–15. 10.17505/jpor.2020.22042 33569148 PMC7842626

